# Predictors of response to neoadjuvant chemo-immunotherapy in metaplastic triple-negative breast cancer

**DOI:** 10.1038/s41523-025-00816-w

**Published:** 2025-10-02

**Authors:** Nour Abuhadra, Fresia Pareja, Charlie White, Yuan Chen, Hannah Wen, Esra Dikoglu, Jinae Park, Stephanie Downs-Canner, Larry Norton, George Plitas, Atif Khan, Sarat Chandarlapaty, Pedram Razavi, Mark Robson, Monica Morrow, Giacomo Montagna

**Affiliations:** 1 Breast Medicine Service, Department of Medicine, Memorial Sloan Kettering Cancer Center, New York, NY USA; 2 Department of Pathology and Laboratory Medicine, Memorial Sloan Kettering Cancer Center, New York, NY USA; 3 Biostatistics Service, Department of Epidemiology and Biostatistics, Memorial Sloan Kettering Cancer Center, New York, NY USA; 4 Breast Service, Department of Surgery, Memorial Sloan Kettering Cancer Center, New York, NY USA; 5 Department of Radiation Oncology, Memorial Sloan Kettering Cancer Center, New York, NY USA

**Keywords:** Breast cancer, Cancer therapy

## Abstract

Metaplastic breast cancer (MpBC) treated with standard chemotherapy has low rates of complete pathological response (pCR)(2–23%). In this study, we evaluate the response to neoadjuvant chemo-immunotherapy (NACI) in early-stage MpBC. Thirty-two stage I-III MpBC patients treated with NACI (KEYNOTE-522 regimen) were prospectively enrolled in an institutional rare tumor program. All MpBC were triple negative; most were of chondromyxoid/matrix-producing (12/32, 38%). The majority had stage II (78%) tumors, 12/32 (37.5%) patients completed NACI, 11/32 (34%) progressed during NACI, and in the remaining 9, NACI was discontinued due to side effects. The pCR rate in the entire cohort was 22% (7/32) and it was statistically higher (5/8, 62%) among patients with high ( ≥ 60%) stromal tumor-infiltrating lymphocytes (sTILs) as compared to patients with < 60% sTILs (1/11, 9%). Most patients received adjuvant systemic therapy (capecitabine 16/32, pembrolizumab 20/32). At a median follow-up of 13 months, there were a total of 2 local recurrences, 10 distant recurrences, and 7 deaths. We demonstrated a modest pCR rate in MpBC with the addition of pembrolizumab (22%). Nonetheless, amongst patients with high sTILs, high pCR rates—comparable to those in the KEYNOTE-522 trial—were observed. These findings suggest that sTILs can be used to triage MpBC patients for NACI.

## Introduction

Metaplastic breast cancer is a rare and aggressive subtype of breast cancer that is defined by the differentiation of glandular epithelium to mesenchymal and/or squamous elements^1^. Metaplastic breast cancer accounts for less than 1% of invasive breast cancers worldwide, and is frequently triple negative, i.e., lacking expression of estrogen receptor, progesterone receptor, and human epidermal growth factor receptor 2 (HER2)^1^. The National Comprehensive Cancer Network (NCCN) guidelines recognize metaplastic histology as an independent, negative prognostic indicator, as patients with metaplastic breast cancer have worse clinical outcomes compared to patients who have non-metaplastic triple-negative breast cancer (TNBC)^2^.

The optimal treatment strategy for patients with early-stage metaplastic breast cancer remains controversial. Retrospective analyses of patients with metaplastic TNBC treated with neoadjuvant chemotherapy (NAC) have demonstrated pathological complete response (pCR) rates of 2% to 11%^3,4^, substantially lower than the 30–54% pCR rates reported for non-metaplastic TNBC^5^, prompting suggestions that patients with metaplastic breast cancer should not receive NAC, or even adjuvant chemotherapy, as standard of care^6^. We have recently shown, in a study where patients with metaplastic breast cancer received neoadjuvant anthracycline- and taxane-based chemotherapy, the pCR rate was 23%^7^. The limited efficacy of standard neoadjuvant chemotherapy (NAC) underscores the critical need to develop more effective therapies for patients with metaplastic breast cancer.

The current treatment algorithm for early-stage TNBC now includes the addition of neoadjuvant and adjuvant immunotherapy based on results from the landmark KEYNOTE-522 trial which showed that the addition of pembrolizumab significantly improved outcomes and increased pCR rates from 56.2% to 63.4% (~13% increase)^8^. Studies have suggested that metaplastic breast cancer, a known chemo-resistant TNBC subtype, may exhibit improved responses with immunotherapy. Indeed, promising clinical responses of metaplastic breast cancer to dual checkpoint blockade in the treatment-refractory setting have been observed, as well as high prevalence of programmed death ligand 1 (PD-L1) expression in this rare subtype^9–11^. Thus, the 2021 U.S. Food and Drug Administration approval of pembrolizumab for early-stage TNBC has rekindled interest for neoadjuvant chemo-immunotherapy (NACI) in the management of metaplastic TNBC. Although up to 95% of metaplastic breast cancers express PD-L1, the benefit of adding immune checkpoint blockade is unknown. We sought to evaluate the response to NACI in early-stage metaplastic breast cancer patients.

## Results

### Patient and tumor characteristics

Patient and clinical characteristics were summarized by pathologic response (Table 1). All patients were female, the median age at diagnosis was 57 years (range, 26–85), 9% self-identified as Asian, 28% self-identified as Black, and 63% self-identified as White. All tumors were triple negative, and the majority of them were poorly differentiated (94%). Median tumor size was 2.9 cm, 78% had stage II disease, and 81% were node negative. Metaplastic subtypes included chondromyxoid/matrix-producing (13/32, 40%), squamous (10/32, 31%), mixed (6/32, 19%), and spindle (3/32, 9%)(Table 1). Germline testing was available in 26 out of 32 patients (6 patients declined testing), and only 2 patients harboured pathogenic mutations in HRD-related genes, specifically CHEK2 (*n* = 2).

**Table 1:** Clinicopathological features of the entire cohort stratified by response to NACI (pathologic complete response versus non-pathologic complete response)

Characteristic	pCR *N* = 7^a^	Residual Disease *N* = 25^a^	*p*-value^b^
Ethnicity			> 0.9
Hispanic or Latino	0 (0%)	2 (8.0%)	
Not Hispanic or Latino	7 (100%)	23 (92%)	
Race			0.2
Asian	0 (0%)	3 (12%)	
Black	4 (57%)	5 (20%)	
White	3 (43%)	17 (68%)	
Menopausal Status			0.6
Post	4 (57%)	18 (72%)	
Pre	3 (43%)	7 (28%)	
Age at diagnosis			0.8
Median (IQR)	52 (48, 63)	57 (50, 62)	
[Range]	[33, 85]	[26, 83]	
Nuclear Grade			> 0.9
II	0 (0%)	2 (8.0%)	
III	7 (100%)	23 (92%)	
Metaplastic Subtype			0.5
Chondromyxoid/Matrix-Producing	4 (57%)	9 (36%)	
Mixed	2 (29%)	4 (16%)	
Spindle	0 (0%)	3 (12%)	
Squamous	1 (14%)	9 (36%)	
Clinical Stage			0.4
I	0 (0%)	4 (16%)	
II	7 (100%)	18 (72%)	
III	0 (0%)	3 (12%)	
T stage at diagnosis			0.6
T1c	2 (29%)	4 (16%)	
T2	5 (71%)	18 (72%)	
T3	0 (0%)	3 (12%)	
N stage at diagnosis			0.10
N0	4 (57%)	22 (88%)	
N1-N2	3 (43%)	3 (12%)	
Ki-67 (cutoff)			> 0.9
< 35	1 (50%)	4 (29%)	
≥ 35	1 (50%)	10 (71%)	
Unknown	5	11	
Completed NACI			0.4
No	3 (43%)	17 (68%)	
Yes	4 (57%)	8 (32%)	
Order of NACI first			0.6
AC	1 (14%)	8 (32%)	
Carbo	6 (86%)	17 (68%)	
RCB Status			
RCB-I	NA	1	
RCB-II	NA	20	
RCB-III	NA	4	
PD-L1 CPS (cutoff - 10)			0.13
< 10	0 (0%)	5 (38%)	
≥ 10	6 (100%)	8 (62%)	
Unknown	1	12	
PD-L1 CPS (cutoff - 1)			> 0.9
< 1	0 (0%)	1 (7.7%)	
≥ 1	6 (100%)	12 (92%)	
Unknown	1	12	
sTIL (cutoff - 20)			0.3
< 20%	0 (0%)	4 (31%)	
≥ 20%	6 (100%)	9 (69%)	
Unknown	1	12	
sTIL (cutoff - 30)			0.3
< 30%	1 (17%)	6 (46%)	
≥ 30%	5 (83%)	7 (54%)	
Unknown	1	12	
sTIL (cutoff - 60)			**0.041**
< 60%	1 (17%)	10 (77%)	
≥ 60%	5 (83%)	3 (23%)	
Unknown	1	12	

*pCR* pathologic complete response, *IQR* interquartile range, *NACI* neoadjuvant chemo-immunotherapy, *AC* doxorubicin and cyclophosphamide, *Pembro* pembrolizumab, *PD-L1* programmed death ligand 1, *CPS* combined positive score, *sTIL* stromal tumor-infiltrating lympohocyte.

^a^n (%).

^b^Fisher’s exact test; Wilcoxon rank sum test.

### Response rates, surgical outcomes, and adjuvant treatment

**Fig. 1:**
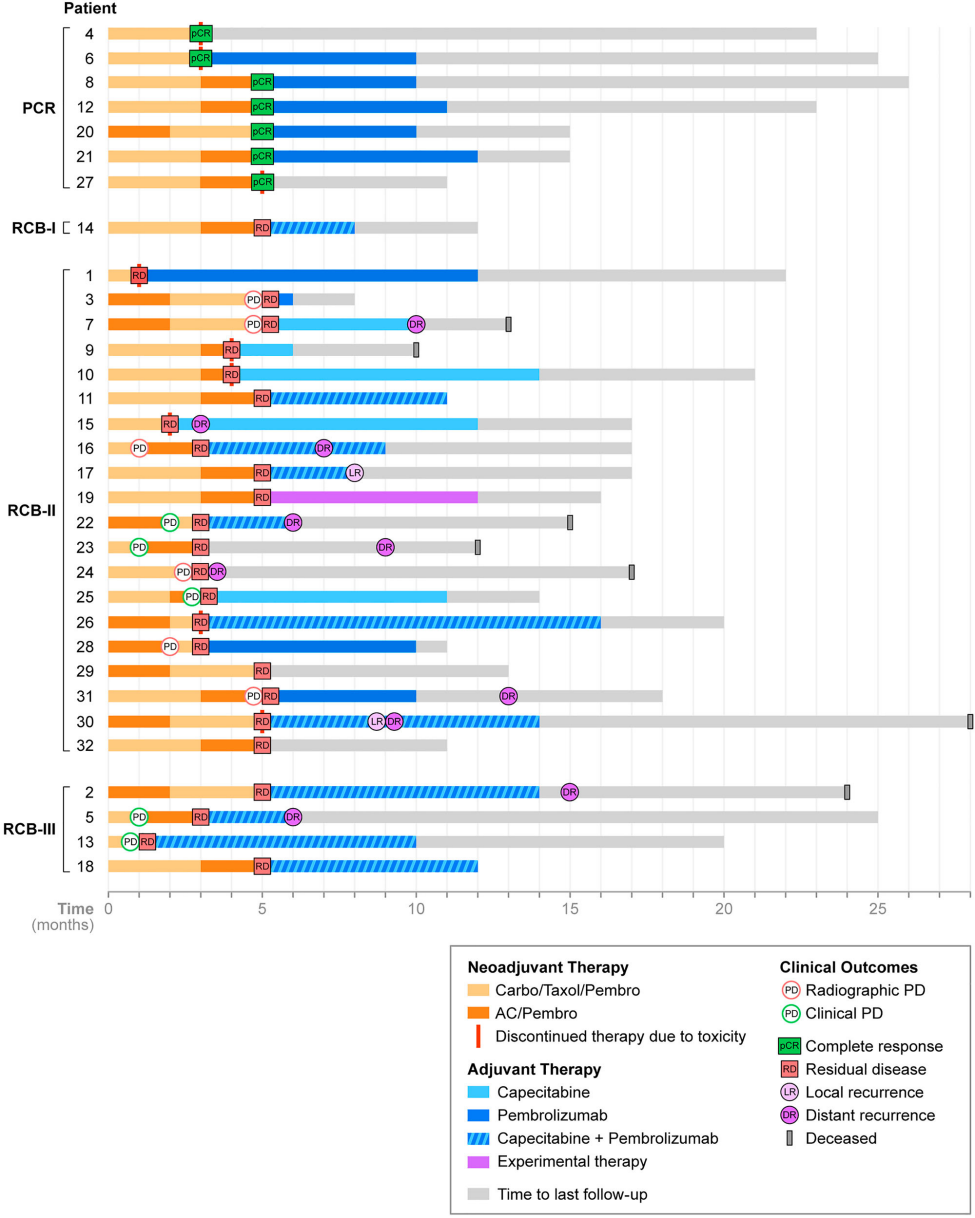
Overview of clinical course. (© 2024 Memorial Sloan-Kettering Cancer Center, Memorial Hospital for Cancer and Allied Diseases, and Sloan-Kettering Institute for Cancer Research, each in New York, NY. All rights reserved. Published with permission.) *pCR* pathologic complete response, *RD* residual disease, *PD* progressive disease, *DR* distant recurrence, *LR* local recurrence, *AC/Pembro* doxorubicin and cyclophosphamide, and pembrolizumab, *Carbo/Taxol/Pembro* carboplatin/ paclitaxel/pembrolizumab.

**Fig. 2:**
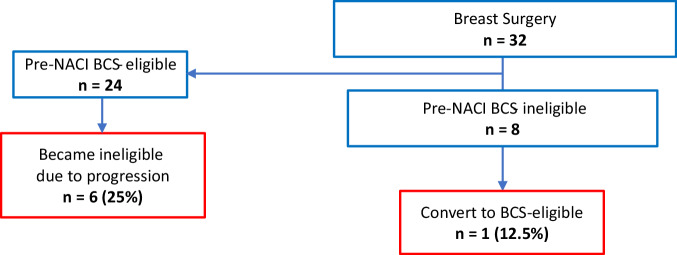
Rate of downstaging to breast-conserving surgery. *NACI* neoadjuvant chemo-immunotherapy, *BCS* breast-conserving surgery.

Twelve patients (37.5%) completed the entire course of NACI; 9 patients discontinued therapy early due to toxicity/patient choice—amongst these patients, only 2 received carboplatin/paclitaxel/pembrolizumab (CbT/Pem) without exposure to anthracycline-based chemotherapy. Eleven patients progressed while on NACI (55% radiographic and 45% clinical); 27% progressed during dose-dense AC/Pem, and 72% progressed during CbT/Pem)(Fig. 1). Despite progression in the latter group, no patients were deemed inoperable, and all patients successfully underwent breast and axillary surgery (17/32, 53% lumpectomy). Among patients deemed ineligible for breast-conservation surgery (BCS) at presentation (8/24), only 1 became BCS-eligible after NACI. Among the 24 patients who were deemed BCS-eligible at presentation, 6 became BCS-ineligible due to progression and had to undergo mastectomy (Fig. 2).

Twenty-six patients were cN0 at presentation, while 6 had biopsy-proven node-positive disease. After NACI, axillary staging was performed with sentinel lymph node biopsy (SLNB) in all cases. Four of 6 node-positive patients successfully converted to ypN0, while the remaining 2 had persistent nodal disease on SLNB and had completion axillary lymph node dissection (ALND). One patient who was clinically node negative at presentation was found to have residual nodal disease at surgery and also underwent ALND. The pCR (ypT0-Tis/ypN0) rate in the entire cohort was 22%. When we evaluated the association between pCR and clinicopathological features (ethnicity, race, menopausal status, nuclear grade, metaplastic subtype, Ki67, PD-L1 [CPS], stromal tumor-infiltrating lymphocyte (sTIL), tumor mutational burden and homologous recombination deficiency-related genes), the only factor significantly associated with pCR was the presence of high stromal sTILs ( ≥ 60%) (*p* = 0.041); PD-L1 expression was not associated with pCR. The relationship between tumor mutational burden and pCR could not be assessed. Of note, sTILs could only be assessed in 19 out of 32 patients (due to insufficient material for analysis). The pCR rate in the high-sTIL group was 62% (*n* = 5/8), whereas the pCR rate in the low-sTIL group was 9% (*n* = 1/11). The only factor associated with pCR was the presence of high sTILs ( ≥ 60%) (*p* = 0.041); of note, we evaluated three different cut-offs for sTILs that have been widely referenced in the literature (20%, 30%, and 60%)(Table 1)^7,12^. The pCR rate in the high sTIL group was 63%.

**Fig. 3:**
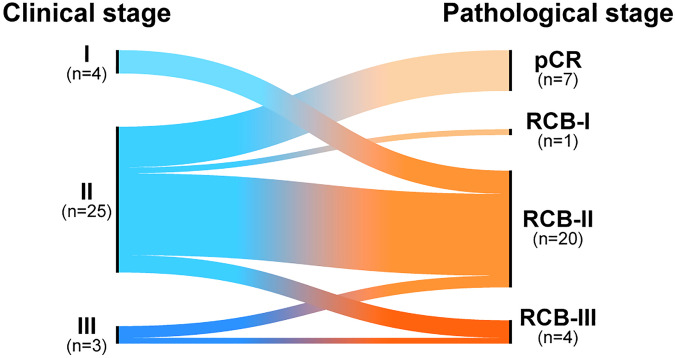
Pathologic complete response by stage at presentation. (© 2024 Memorial Sloan-Kettering Cancer Center, Memorial Hospital for Cancer and Allied Diseases, and Sloan-Kettering Institute for Cancer Research, each in New York, NY. All rights reserved. Published with permission.) *pCR*, pathologic complete response, *RCB*, residual cancer burden.

Notably, only 1 out of 11 patients with squamous subtype of MpBC achieved pCR (9%), and 4 out of 7 patients with pCR had MpBC of the chondromyxoid/matrix-producing subtype (57%). None of the patients with clinical stage I or III disease achieved pCR, although this should be interpreted with caution, given very small representative numbers (Fig. 3). The majority of patients went on to receive adjuvant systemic therapy (81%). Amongst patients with pCR, 5/7 patients completed adjuvant pembrolizumab per standard of care. Amongst the 25 patients with residual disease, 3 patients who did not complete NACI went on to receive chemotherapy (i.e., AC/Pem or CbT/Pem) in the adjuvant setting. Sixteen of 25 patients received adjuvant capecitabine, and 20 of 25 patients received adjuvant pembrolizumab per standard of care. Two patients were enrolled on clinical trials for post-NAC residual disease, and two patients received adjuvant aromatase inhibitors for low estrogen receptor/progesterone receptor expression ( < 10%) following adjuvant capecitabine +/- pembrolizumab. The majority of patients completed adjuvant radiation (93%).

### Oncological outcomes

**Fig. 4:**
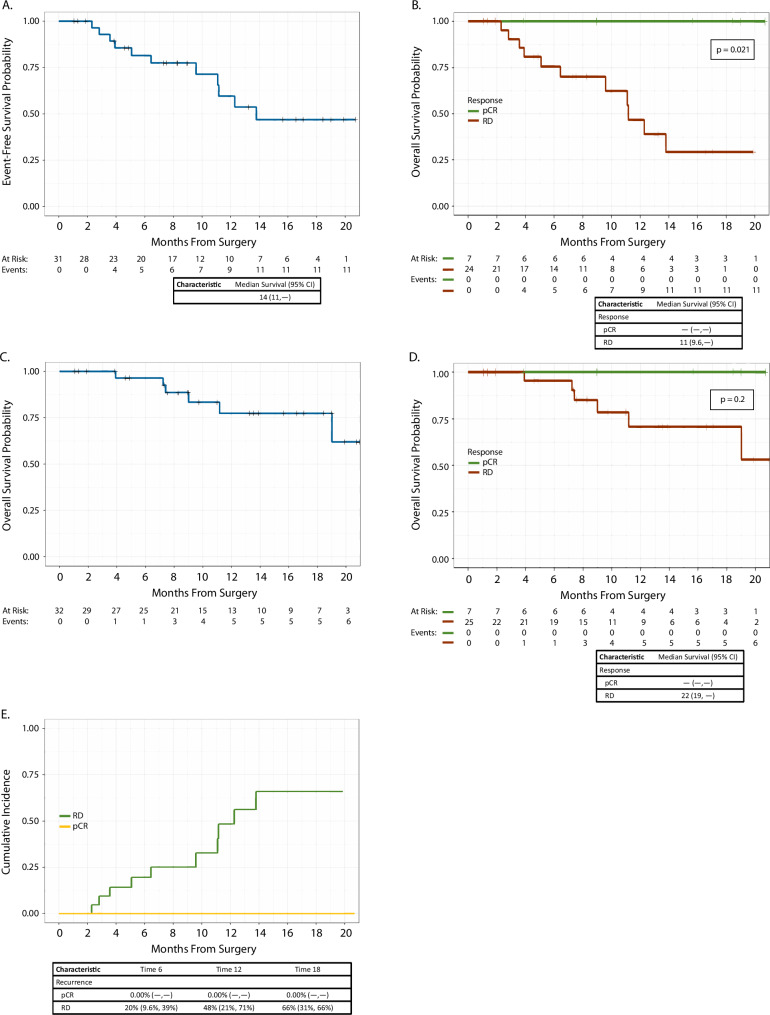
Event-free survival and overall survival Kaplan-Meier curves stratified by type of response to neoadjuvant chemo-immunotherapy. **A** Event-free survival in entire cohort; **B** event-free survival by response; **C** overall survival in entire cohort; **D** overall survival by response; and **E** cumulative incidence of recurrence by response. *CI* confidence interval, *pCR* pathologic complete response, *RD* residual disease.

At a median follow-up of 13 months (95% confidence interval [CI] 8.9–13), there were 2 local recurrences, 10 distant recurrences, and 7 deaths on study. Specifically, amongst patients with pCR (*n* = 7), none have experienced a local or distant recurrence. Of important note, the event-free survival was significantly improved amongst patients with pCR (*p* = 0.021)(Fig. 4B) with a trend toward improvement in overall survival, although with relatively short-term follow-up at this time. At 18 months, 66% of patients with residual disease experienced a recurrence compared to 0% of patients with pCR. Amongst patients with residual disease (*n* = 25), 2 experienced a local recurrence and 10 experienced a distant recurrence within 1 year of completing adjuvant systemic therapy. The majority of distant recurrences occurred while still on adjuvant systemic therapy (70%). Critically, the most common sites of distant recurrence were the lung (70%) and lymph nodes (30%); the majority of patients received sacituzumab govitecan as first-line therapy (60%).

## Discussion

Immune checkpoint blockade combined with NAC has improved pCR rates in patients with stage II-III TNBC. Metaplastic breast cancer is a histologic TNBC subtype historically associated with poor response to NAC (2–23%). To date, there have been no studies reporting on the clinical and pathologic features associated with response in patients with metaplastic breast cancer receiving neoadjuvant immune checkpoint blockade. Furthermore, there are no long-term outcome data on metaplastic TNBC treated with neoadjuvant immune checkpoint blockade. In this prospective study evaluating neoadjuvant immune checkpoint blockade in metaplastic breast cancer, we demonstrate modest pCR rates in metaplastic breast cancer with the addition of pembrolizumab to chemotherapy (22%). In retrospective studies, there have been pCR rates as low as 0% reported; however, in one prospective evaluation of a patient cohort uniformly treated with dose-dense AC-T, the pCR rate was comparable to what we report here (23%)^7^. Interestingly, upon our evaluation of clinical and pathological factors associated with pCR, we identified that high sTILs ( ≥ 60%) were associated with improved pCR rates. Amongst patients with high sTILs ( ≥ 60%), the pCR rate was 63% (*n* = 5/8) which is comparable to the rates reported in the final analysis of KEYNOTE-522, although this should be interpreted with caution in small subsets^8^. Multiple studies have found that metaplastic breast cancer frequently expresses PD-L1^11,13,14^ and tends to have higher sTIL incidence. A recent series identified that overall, 34% of its metaplastic breast cancer patients had intermediate or high sTIL infiltration; interestingly, this series also found that intermediate or high sTILs, but not PD-L1 positivity, correlated with improved clinical outcomes^15^.

Furthermore, 4 out of 6 cN+ patients successfully converted to ypN0 (with treatment effect present in 2 out of 4 cases)—although nodal involvement is historically considered uncommon in metaplastic breast cancer, in our entire cohort, 19% of patients were clinically node positive at diagnosis, and avoidance of completion axillary dissection represents a significant clinical outcome that greatly impacts patient quality of life. While NACI is generally an effective way to avoid mastectomy^16^, in this cohort, the conversion from BCS-ineligible to BCS-eligible was low (only 1 patient avoided mastectomy), while a quarter of patients initially eligible for BCS had to have a mastectomy due to progression during NACI.

The KEYNOTE-522 regimen introduced the addition of carboplatin as well as immune checkpoint blockade to our standard of care. It is well-established that tumors with HRD are more sensitive to DNA-damaging chemotherapy such as platinum. There has also been some data to suggest a relationship between metaplastic breast cancer and HRD-related alterations^17^. In our cohort, only 2 patients harboured pathogenic germline alterations in HRD-related genes, so this relationship could not be assessed. This warrants further investigation in a larger cohort of patients.

Metaplastic breast cancer is associated with significantly worse survival outcomes compared to other types of breast cancer; literature on the prognostic implications of a pCR in metaplastic breast cancer after exposure to immunotherapy is lacking. One study evaluated the prognostic implications of pCR following NAC alone, demonstrating a 5-year overall survival difference amongst metaplastic breast cancer patients with pCR versus residual disease that was statistically significant (93 versus 63%, *p* < .001)^18^. Ours is the first study to demonstrate significant improvement in event-free survival amongst metaplastic breast cancer patients with pCR following NACI (at 18 months, all patients with pCR are alive without evidence of local or distant recurrence). The majority of patients on this study completed appropriate adjuvant therapy (81%); unfortunately, despite appropriate adjuvant treatment, amongst patients with residual disease, 20% of patients experienced a recurrence at 6 months, 48% of patients experienced a recurrence at 12 months, and 66% experienced a recurrence at 18 months (despite adjuvant treatment) (Fig. 4E). This highlights an urgent, unmet need for better treatment strategies in this post-NACI residual disease setting, and screening for clinical trials represents an important strategy in this regard. There are several ongoing clinical trials evaluating various agents, with a special focus on antibody-drug conjugates, such as sacituzumab govitecan and datopotamab deruxtecan, in combination with immunotherapy in this space (ClinicalTrials.gov ID Nos. NCT04595565; NCT05629585; and NCT05633654).

**Fig. 5:**
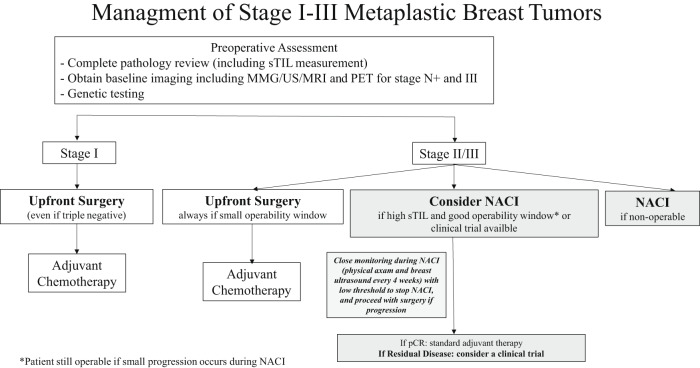
Management of stage I-III metaplastic triple negative breast cancers. *sTILs* stromal tumor-infiltrating lymphocytes, *MMG* mammogram, *US* ultrasound, *PET* positron emission tomography, *NACI* neoadjuvant chemo-immunotherapy, *pCR* pathologic complete response.

This work suggests that NACI should be considered in metaplastic breast cancer with close monitoring, and that, notably, sTILs may be used to select metaplastic breast cancer patients for NACI. Interestingly, none of the patients with clinical stage I or III disease in our study achieved pCR (Fig. 3). Although the patient population is small, and although our findings need to be confirmed in larger series, we suggest that the risks of adding immunotherapy in patients with stage I disease may not outweigh the benefits—this is further supported by our finding that none of the stage I patients have experienced a local or distant recurrence despite having residual disease at time of surgery. This suggests that clinical stage I MpBC patients may have a favourable prognosis and do not require escalating treatment with NACI. Conversely, in patients with stage III disease, upfront surgery may be favored if their window of operability is small (Fig. 5). Close monitoring with ultrasound was strongly encouraged on this protocol—amongst patients with progression, no patients were deemed inoperable, and all patients successfully underwent surgery. This highlights the need for close clinical and radiographic monitoring of patients when considering NACI in metaplastic breast cancer. Notably, 2 patients achieved pCR with CbT/Pem alone (treatment discontinued due to toxicity), suggesting a subset of “super-responders” in this patient population. There have been a few cases reported in the literature of dramatic responses to immunotherapy in metaplastic breast cancer^9,19^, highlighting an urgent need to identify biomarkers of response in this disease biology. However, 35% of patients progressed during NACI despite polychemotherapy and immunotherapy, highlighting the need for better treatment strategies in this treatment-refractory subset of metaplastic breast cancer. The dichotomy in responses to immune checkpoint blockade (super-responders versus those patients in the treatment-refractory subset) may suggest the presence of unique biomarkers for immune response in metaplastic breast cancer. Translational work is ongoing utilizing spatial transcriptomics to identify differences in the immune microenvironment between these subgroups.

Our study has limitations, such as its small patient population. Moreover, the biospecimen availability for select patients was limited by either insufficient material for analysis for PD-L1/sTILs or by consent parameters (i.e., patients who consented to the use of their clinical data but who declined the use of their biopsy specimens for research purposes). Nonetheless, this is the largest prospective evaluation of response to NACI in metaplastic breast cancer to date, and in the absence of known metaplastic breast cancer patients in the KEYNOTE-522 study, it is the first to identify a possible clinicopathologic predictor of response in metaplastic breast cancer. Our study shows that although metaplastic breast cancer shows a modest pCR rate in response to chemo-immunotherapy, the subset of cases with high sTILs exhibit higher pCR rates, and that—in what would represent a significant advance in the management and treatment of this rare and aggressive breast cancer subtype—sTIL assessment could be used to triage metaplastic breast cancer patients for NACI.

## Methods

### Patient characteristics

This cohort consists of 32 stage I-III metaplastic breast cancer patients who were treated with neoadjuvant KEYNOTE-522 between July 2021 and January 2024 as part of the Memorial Sloan Kettering Cancer Center Rare Breast Cancer Program (CARE-4-RARE), a structured clinical and translational program whose primary aim is to identify biomarkers of response and/or resistance to systemic therapies in patients with metaplastic breast cancer and other rare types of breast cancer. All clinical data, pathological data, and patient specimens were collected under the Memorial Sloan Kettering CARE-4-RARE Institutional Review Board protocol (IRB No. 22–213) in accordance with the Declaration of Helsinki, and informed consent to participate in this study was obtained from participants.

### Clinical and imaging surveillance

At presentation, patients underwent breast imaging including mammogram, ultrasound, and MRI. Before starting NACI, patients with abnormal axillary nodes on physical exam or on imaging underwent fine needle aspiration or core biopsy of the abnormal nodes. During NAC, all patients underwent close clinical and imaging monitoring, with physical exams performed by both the medical oncologist and the surgeon every 4–6 weeks. Close monitoring with ultrasound was strongly encouraged on this protocol (Timepoint No. 1: after 2 cycles of CbT/Pem; Timepoint No. 2: prior to initiating dose-dense doxorubicin and cyclophosphamide, and pembrolizumab (AC/Pem); and Timepoint No. 3: after 2 cycles of dose-dense AC/Pem). Tumor progression was defined as a ≥ 20% increase in the sum of the longest diameter of the tumor according to the RECIST criteria^20^.

### Tissue processing and immunohistochemistry

Estrogen receptor, progesterone receptor, and HER2 status were determined according to American Society of Clinical Oncology/College of American Pathologists (ASCO/CAP) guidelines^21,22^, and retrieved from medical chart analysis. Low estrogen receptor/progesterone receptor ( < 10%) patients were included in this analysis. Metaplastic breast cancer histology was defined using the criteria set by the World Health Organization^23^. Extent of tumor-infiltrating lymphocyte (TIL) infiltration was assessed on baseline core biopsies according to the International TIL Working Group guidelines^12^. pCR was defined as the absence of invasive cancer within the breast and regional lymph nodes (ypT0/ypN0 or ypTis/ypN0), and residual disease was defined as the lack of pCR as per the American Joint Committee on Cancer^5^. Residual cancer burden indices and class were calculated according to previously published methods from the histopathological features of residual disease specimens after NACI^24^. PD-L1 expression was assessed using the VENTANA SP263 assay. The combined positive score was defined as the sum of PD-L1-expressing tumor cells, lymphocytes, and macrophages divided by the total number of tumor cells multiplied by 100^25,26^.

### Statistical methods

Continuous and categorical variables were compared by response status using the Wilcoxon rank sum test and Fisher’s exact test, respectively. Event-free survival was defined as time from surgery to local/distant recurrence or death. Overall survival was defined as time from surgery to death. Alive patients were censored at last follow-up. Time to recurrence was defined as time from surgery to first local/distant recurrence, where death without recurrence was treated as a competing event. Event-free survival and overall survival were estimated using the Kaplan-Meier method. The cumulative incidence function was used to estimate time to recurrence. One patient had a distant recurrence prior to surgery; this patient was excluded from event-free survival and time-to-recurrence analyses.

## Data Availability

The datasets used and/or analyzed during the current study are available from the corresponding author on reasonable request.

## Code availability

No custom computer code or scripts were used for this study. The underlying code for this study (and training/validation datasets) is not publicly available, but may be made available to qualified researchers on reasonable request from the corresponding author.
